# Low seroprevalence of *Coxiella burnetii* in Boer goats in Missouri

**DOI:** 10.1186/1756-0500-7-421

**Published:** 2014-07-04

**Authors:** Molly D Baker, Patrick O Pithua

**Affiliations:** 1 Department of Veterinary Medicine & Surgery, Veterinary Medicine Teaching Hospital, College of Veterinary Medicine, University of Missouri, 900 E. Campus Drive, Columbia, MO 65211, USA

**Keywords:** *Coxiella burnetii*, Goats, Q fever, Missouri, United States

## Abstract

**Background:**

Goats are known reservoirs of *Coxiella burnetii,* the etiologic agent of Q fever. However, there has been very little research on the prevalence of *C. burnetii* exposure and risk in meat goats farmed in the US. Banked serum samples were secondarily tested for *C. burnetii* specific antibodies.

**Findings:**

The animal and herd-level seroprevalence estimates for *C. burnetii* were 1.2% (3/249) and 4.2% (1/24) respectively. Within-herd seroprevalence ranged from 0% to 1.2%.

**Conclusions:**

This study indicates that seroprevalence of *C. burnetii* in Boer goats raised in Missouri was low, but it does not preclude the existence of a higher level of infection in Missouri’s meat goat herds. This result is inconclusive because this study was disadvantaged by the small number of individual animal and herds tested, which compromised the statistical power of this study to detect a possible higher seroprevalence of *C. burnetii* in this population, if present. More research is warranted to corroborate the preliminary findings reported here in order to determine the public health significance *C. burnetii* infection risks associated with contemporary goat production systems in the US.

## Findings

### Background

*Coxiella burnetii*, an obligate intracellular pathogen of both humans and animals is the causative agent of Q-fever [1]. Zoonotic Q fever in humans has generally been linked with transitory outbreaks of the disease in animals [2,3]. Humans are primarily infected through inhalation of *C. burnetii* in aerosols, resulting from occupational exposures, although transmissions through oral ingestion of contaminated food are documented [4,5]. While infected ruminants remain largely asymptomatic, the primary signs of *C. burnetii* include spontaneous, late-term abortions in pregnant animals. There is evidence that *C. burnetii* is a public health hazard in the US with humans being exposed to the bacteria through milk. A recent report found that 94% of bulk tank milk samples collected from US dairy herds contained *C. burnetii* specific DNA [6]. Loftis and others detected *C. burnetii* in 42.9% (9/21) of commercial raw milk samples in the US [7], and a recent case report found Q fever clusters among raw milk consumers in the State of Michigan [4].

Small ruminants and goats, in particular, are primary reservoirs of *C. burnetii*[8]. A review of the burden of Q fever in the US revealed a 41.6% average prevalence of *C. burnetii* infection in goats [9]. Studies in sheep in Wyoming revealed a 7% seroprevalence of *C. burnetii*[10]. Recent Studies in California [9,11] and Washington [12] suggested that *C. burnetii* was endemic in US meat and milk goats, although its prevalence may be underestimated and the overall distribution of the disease in the US remains unknown [9,11,12]. No studies have evaluated the prevalence and risk factors of *C. burnetii* infections in meat goats farmed in Missouri. The aim of this pilot investigation was to determine whether Boer goats, the preponderant meat goat breed in Missouri, were exposed to *C. burnetii* and to estimate seroprevalence of the exposure.

### Methods

There are approximately 3,955 goat herds in Missouri with an estimated 103,669 goats [13]. Based on the 2012 agricultural census data, Boer goats are a dominant meat breed accounting for over 86% of all goats in Missouri [13]. In this study, banked serum samples from a previous study performed to estimate the apparent prevalence of *Mycobacterium avium* subsp. *paratuberculosis* (MAP) in Missouri Boer goat herds were secondarily tested for *C. burnetii* specific antibodies [14]. Participation in the original study was voluntary and protocols were approved by the Institutional Animal Use and Care Committee at the University of Missouri (Protocol # 7395). Herds and animals were selected based on criteria described elsewhere [14]. Briefly, Boer goat herds in the State of Missouri constituted the sampling unit, and the target population included goats 24 months of age and older. Herds containing other breeds of goats were excluded. Sixty-one herds were required to estimate seroprevalence of at least 20% for MAP, with an allowable error of 10% and a 5% type I error rate. Twenty-five (41%) herds of the required 61 herds were ultimately enrolled due to low farmer response rates.

Herd visits for sample collection were completed between May and September, 2012, and all eligible animals (i.e. Boer goats ≥ 24 months old) present in herds that agreed to the study were tested. The mean ± SD (min., max.) number of animals sampled per herd was 25 ± 19(2,57) [14]. The number of herds sampled that had < 50, 50–100, and 100–200 goats were 19 (76%), 5 (20%), and 1 (4%) respectively [14].

Blood samples were collected via jugular venepuncture from a total of 629 goats, centrifuged at 3,000 *g* and the resulting sera stored at -80°C. Only a subset of these banked serum samples was randomly selected and tested for *C. burnetii* due to budgetary constraints. Of the 629 sera available, 249 were submitted to the National Veterinary Services Laboratories in Ames, Iowa, for processing and testing. These samples were tested for *C. burnetii* antibodies using a commercial ELISA kit following the manufacturer’s guidelines (IDEXX Switzerland AG, Liebelfld-Bern, Switzerland). The kit consisted of micro titre plate wells laced with phase I and II inactivated *C. burnetii* antigens from the Nile Mile strain on which Q fever specific antibodies bind [15]. Plates were washed to remove unbound antibodies. A peroxidase-labeled anti-ruminant immunoglobulin G was added to the *C. burnetii* antigen-antibody complex in each well. The micro titre wells were incubated and then washed to remove unbound reagents. The optical density (OD) value of each sample was measured by a spectrophotometer (CHEKIT Q Fever Antibody ELISA, IDEXX Switzerland AG, Liebelfld-Bern, Switzerland). Seropositivity was determined as a percentage of test samples’ OD value relative to the positive control, and corrected for the OD values from the negative control. Test samples with OD values < 30% were considered negative, samples with OD values between 30% and 40% were reported as suspect, and those with OD values ≥ 40% were declared positive for Q-fever antibodies. The animal level, within-herd, and between-herd seroprevalence were calculated by dividing the number of test positive outcomes by the appropriate denominator for each measure. Confidence intervals (95%) for the estimated seroprevalence were calculated using the Wilson binomial approximation method as described [16].

### Results and discussion

In total, 249 serum samples were tested. Ninety-eight percent (244/249) of these were negative (OD < 30%), 0.8% (2/249) were suspects (≥30% OD ≤ 40%), and 1.2% (3/249) were positive for *C. Burnetii* antibodies. Based on test results, animal-level, herd-level and within-herd (range) seroprevalence estimates were 1.2% (95% CI = 0.4%-3.5%), 4.2% (95% = 0.7%-20.2%), and 0% to 1.2%, respectively. This estimate was lower than previously reported in California dairy goats (i.e. 24%) [11]. Similarly, the apparent prevalence estimates in this study were lower than the 8% (144/1794) animal-level; 8.6% (9/105) herd-level; and 2.9% to 75.8% within-herd level estimates for *C. burnetii* seroprevalence reported in a recent study of domestic goats in Washington [12].

In the current study, only Boer goats (i.e., a meat breed) were tested. In contrast, the Washington study focused on goats of all production types (i.e. meat and dairy goats) while only dairy goats were studied in California [11,12]. Thus, the observed disparity in *C. burnetii* seroprevalence between the current study and those performed in Washington, and California was likely a function of breed (or production types) predisposition to *C. burnetii* infection with apparent risks being greater for dairy breeds of goats than meat breeds (i.e. Boer goats). However, while the most-recent large-scale *C. burnetii* outbreaks in humans in The Netherlands were linked to dairy goat farms as the source [3], no plausible explanation exists in support of the breed-susceptibility hypothesis. Increased levels of exposure, due to a high within-herd *C. burnetii* prevalence rather than genetic or breed predisposition, is a more likely reason for the apparently higher prevalence of infection in the dairy breeds seen in California [11]. The low *C. burnetii* seroprevalence observed in this study could also be due to differences in the mobility patterns of dairy versus meat goats. For instance, in dairy goat management, goats may be moved to other farms over their lifetime as owners’ buy-in or sell-out animals as herd replacements [2]. Between-herds mobility is a well-recognized risk factor for infectious disease transmission in livestock and is likely a more common phenomenon in dairy versus meat goats hence the higher animal and herd seroprevalence of *C. burnetii* observed in dairy relative to the meat breeds of goats [2].

It is possible that the higher prevalence of *C. burnetii* observed in dairy goat breeds may be the consequences of differences in management between the two production types. Goats for meat in the US are raised mainly under an extensive or grazing system [17]. Infectious disease such as C*. burnetii* likely spreads faster under a confinement system of production. The latter is typical for most dairy goats in the US.

The current study was similar to the Washington survey in the use of an ELISA with phase I and II antigens from the Nile Mile strain of *C. burnetii* to detect antibodies in sera [12,15]. Moreover, in both studies only goats from herds with no known history of *C. burnetii* infections were tested. However, the difference in the *C. burnetii* seroprevalence observed between Missouri, and California may have been due to the difference in diagnostic tests used, given that sera from dairy goats in California were tested using a micro agglutination assay containing phase II antigens from the C76 *C. burnetii* strain [11].

Though a recent outbreak of Q fever in humans was linked to a single dairy goat farm in Washington State [2], underscoring the significance of goat associated zoonotic risk of *C. burnetii* in the US, to date, risk factors for the emergence of *C. burnetii* in US goat populations have not been fully studied. A recent study in The Netherlands found an association between large flock size (i.e., ≥ 800 goats), proximity to infected herds (i.e., < 8 Km), high cattle density in the flock district (≥800 cattle/Km^2^), presence of cats or dogs in the goat pens, and the use of imported straw, and *C. burnetii* seroprevalence in commercial dairy goat farms [18]. However, the epidemiology of *C. burnetii* infections in goats in The Netherlands may not reflect the epidemiology of the disease in the US caprine population given differences in goat productions systems. To better understand the human health risk of *C. burnetii* infection associated with contemporary goat production systems in the US, and to develop appropriate interventions to limit occupational exposure and public health risks, knowledge of prevalence, distribution, and risk factors for *C. burnetii* infections in meat goats are required.

A disadvantage of this study was that it was impossible to ascertain the counties and therefore, the geographic scope of the disease in Missouri represented in the samples tested because of their secondary nature. Additionally, the relatively few goats tested in this study may have undermined the statistical power of this study to detect a larger number of exposed animals hence the low seroprevalence observed. It entirely is possible that the seroprevalence of *C. burnetii* in Boer goats in Missouri are higher than could be demonstrated with the current data. The results reported here is inconclusive. We contend that if the animal and herd level apparent prevalence of *C. burnetii* in Boer goats in Missouri are as high as reported in California or Washington [11,12], then future studies in Missouri will require a state-wide representative sample of ≥ 984 goats from at least 453 herds to estimate an animal-level seroprevalence ≥ 20% or a herd-level apparent prevalence ≥ 8% with 95% confidence level, assuming an infinite population of goats in Missouri.

### Conclusions

To our knowledge, this is the first study of *C. burnetii* seroprevalence in Missouri Boer goat herds. While this study found a low seroprevalence of *C. burnetii* in a limited sample of Boer goats from Missouri, the results do not preclude the existence of infection in Missouri’s Boer goat herds. The results shown here is likely indicative of a naïve population of goats, which if sufficiently exposed to animals actively shedding *C. burnetii*, could acquire prevalence as high if not higher than previously reported in the US [9,11,12]. This study was limited by the small number of individual animal and herds tested. This may have compromised the statistical power of this study to detect a possible higher seroprevalence. There is a need for large-scale surveillance studies to determine the underlying risk factors for emergence and transmission of *C. burnetii* in this rapidly growing US livestock sector.

## Competing interests

The authors declare that they have no competing interests.

## Authors’ contributions

MDB, a summer research scholar, participated in the design of the study and performed the statistical analysis. PP conceived of the study, provided the samples, and participated in its design and coordination and helped to draft the manuscript. All authors read and approved the final manuscript.
